# Supplementary material for: A study on the measurement of inter-provincial trade costs in Yangtze River Delta from the perspective of value-added trade and its promotion effect
Source: PLoS One. 2024 Mar 8;19(3):e0295654. doi: 10.1371/journal.pone.0295654 (PMC10923410; doi:10.1371/journal.pone.0295654)
Supplement: Attachment — Submitted filename: PONE-D-23-25349_reviewer (Sept.23).pdf [file pone.0295654.s001.pdf]

PLOS ONE

A Study on the Measurement of Inter-provincial Trade Costs in Yangtze River Delta from the Perspective of Value Added Trade and its Promotion Effect

--Manuscript Draft--

**Manuscript Number:**	PONE-D-23-25349
**Article Type:**	Research Article
**Full Title:**	A Study on the Measurement of Inter-provincial Trade Costs in Yangtze River Delta from the Perspective of Value Added Trade and its Promotion Effect
**Short Title:**	the Measurement of Inter-provincial Trade Costs in Yangtze River Delta
**Corresponding Author:**	YANG WANG<br>Shanghai Dianji University<br>Shanghai, CHINA
**Keywords:**	value-added trade; inter-provincial trade costs; integrated development; promotion effect
**Abstract:**	This paper measures the inter-provincial trade costs in the Yangtze River Delta (YRD) using the Novy (2013) model with improved value-added trade data and uses the differential decomposition method to explore the promotion effect of inter-provincial trade costs on inter-provincial trade development in the YRD. The results show that the inter-provincial trade costs of the provinces/municipalities in the Yangtze River Delta have increased and decreased, but the changes are small, and there are significant differences in sectoral and bilateral trade costs; the results of the differential decomposition show that the contribution of inter-provincial trade costs to inter-provincial trade development in the Yangtze River Delta is much lower than that of economic growth. Therefore, the Yangtze River Delta should deepen the division of labor and cooperation, give full play to the radiation and leading role of Shanghai as an economic center, and accelerate the digital innovation transformation of the service industry to reduce inter-provincial trade costs and achieve a higher level of integrated development of the Yangtze River Delta.
**Order of Authors:**	RENXIANG LU
	YANG WANG
	HUANYUE TONG
**Additional Information:**	
**Question**	**Response**
**Financial Disclosure**<br><br>Enter a financial disclosure statement that describes the sources of funding for the work included in this submission. Review the submission guidelines for detailed requirements. View published research articles from *PLOS ONE* for specific examples.<br><br>This statement is required for submission and **will appear in the published article** if the submission is accepted. Please make sure it is accurate.	The author(s) received no specific funding for this work.

Powered by Editorial Manager® and ProduXion Manager® from Aries Systems Corporation

**Unfunded studies**<br>Enter: *The author(s) received no specific funding for this work.*<br><br>**Funded studies**<br>Enter a statement with the following details:<br>• Initials of the authors who received each award<br>• Grant numbers awarded to each author<br>• The full name of each funder<br>• URL of each funder website<br>• Did the sponsors or funders play any role in the study design, data collection and analysis, decision to publish, or preparation of the manuscript?<br>• **NO** - Include this sentence at the end of your statement: *The funders had no role in study design, data collection and analysis, decision to publish, or preparation of the manuscript.*<br>• **YES** - Specify the role(s) played.<br><br>* typeset	
**Competing Interests**<br><br>Use the instructions below to enter a competing interest statement for this submission. On behalf of all authors, disclose any competing interests that could be perceived to bias this work—acknowledging all financial support and any other relevant financial or non-financial competing interests.<br><br>This statement is required for submission and **will appear in the published article** if the submission is accepted. Please make sure it is accurate and that any funding sources listed in your Funding Information later in the submission form are also declared in your Financial Disclosure statement.<br><br>View published research articles from *PLOS ONE* for specific examples.	The authors have declared that no competing interests exist.

**NO authors have competing interests**<br><br>Enter: *The authors have declared that no competing interests exist.*<br><br>**Authors with competing interests**<br><br>Enter competing interest details beginning with this statement:<br><br>*I have read the journal's policy and the authors of this manuscript have the following competing interests: [insert competing interests here]*<br><br>* typeset	
**Ethics Statement**<br><br>Enter an ethics statement for this submission. This statement is required if the study involved:<br><br>• Human participants<br>• Human specimens or tissue<br>• Vertebrate animals or cephalopods<br>• Vertebrate embryos or tissues<br>• Field research<br><br>Write "N/A" if the submission does not require an ethics statement.<br><br>General guidance is provided below. Consult the submission guidelines for detailed instructions. **Make sure that all information entered here is included in the Methods section of the manuscript.**	N/A

**Format for specific study types**<br><br>**Human Subject Research (involving human participants and/or tissue)**<br>• Give the name of the institutional review board or ethics committee that approved the study<br>• Include the approval number and/or a statement indicating approval of this research<br>• Indicate the form of consent obtained (written/oral) or the reason that consent was not obtained (e.g. the data were analyzed anonymously)<br><br>**Animal Research (involving vertebrate animals, embryos or tissues)**<br>• Provide the name of the Institutional Animal Care and Use Committee (IACUC) or other relevant ethics board that reviewed the study protocol, and indicate whether they approved this research or granted a formal waiver of ethical approval<br>• Include an approval number if one was obtained<br>• If the study involved *non-human primates*, add *additional details* about animal welfare and steps taken to ameliorate suffering<br>• If anesthesia, euthanasia, or any kind of animal sacrifice is part of the study, include briefly which substances and/or methods were applied<br><br>**Field Research**<br><br>Include the following details if this study involves the collection of plant, animal, or other materials from a natural setting:<br>• Field permit number<br>• Name of the institution or relevant body that granted permission	
**Data Availability**<br><br>Authors are required to make all data underlying the findings described fully available, without restriction, and from the time of publication. PLOS allows rare exceptions to address legal and ethical concerns. See the PLOS Data Policy and FAQ for detailed information.	Yes - all data are fully available without restriction

A Data Availability Statement describing where the data can be found is required at submission. Your answers to this question constitute the Data Availability Statement and **will be published in the article**, if accepted.<br><br>**Important:** Stating 'data available on request from the author' is not sufficient. If your data are only available upon request, select 'No' for the first question and explain your exceptional situation in the text box.<br><br>Do the authors confirm that all data underlying the findings described in their manuscript are fully available without restriction?	
**Describe where the data may be found in full sentences. If you are copying our sample text, replace any instances of XXX with the appropriate details.**<br><br>• If the data are **held or will be held in a public repository**, include URLs, accession numbers or DOIs. If this information will only be available after acceptance, indicate this by ticking the box below. For example: *All XXX files are available from the XXX database (accession number(s) XXX, XXX.).*<br>• If the data are all contained **within the manuscript and/or Supporting Information files**, enter the following: *All relevant data are within the manuscript and its Supporting Information files.*<br>• If neither of these applies but you are able to provide **details of access elsewhere**, with or without limitations, please do so. For example:<br><br>*Data cannot be shared publicly because of [XXX]. Data are available from the XXX Institutional Data Access / Ethics Committee (contact via XXX) for researchers who meet the criteria for access to confidential data.*<br><br>*The data underlying the results presented in the study are available from (include the name of the third party*	All data files are available from the Input-output table between provinces and cities in China.

and contact information or URL).<br>• This text is appropriate if the data are owned by a third party and authors do not have permission to share the data.<br><br>* typeset	
Additional data availability information:	

Manuscript Click here to access/download;Manuscript;20230721.docx

A Study on the Measurement of Inter-provincial Trade Costs in Yangtze River Delta from the Perspective of Value Added Trade and its Promotion Effect

Lu-Renxiang[1]; Wang Yang[1*]; Tong-Huanyue

[1] School of Business, Shanghai Dianji University, Shanghai, China

[*] Corresponding author's e-mail: yangwang@sdju.edu.cn

Abstract: This paper measures the inter-provincial trade costs in the Yangtze River Delta (YRD) using the Novy (2013) model with improved value-added trade data and uses the differential decomposition method to explore the promotion effect of inter-provincial trade costs on inter-provincial trade development in the YRD. The results show that the inter-provincial trade costs of the provinces/municipalities in the Yangtze River Delta have increased and decreased, but the changes are small, and there are significant differences in sectoral and bilateral trade costs; the results of the differential decomposition show that the contribution of inter-provincial trade costs to inter-provincial trade development in the Yangtze River Delta is much lower than that of economic growth. Therefore, the Yangtze River Delta should deepen the division of labor and cooperation, give full play to the radiation and leading role of Shanghai as an economic center, and accelerate the digital innovation transformation of the service industry to reduce inter-provincial trade costs and achieve a higher level of integrated development of the Yangtze River Delta.

Keywords: value-added trade; inter-provincial trade costs; integrated development; promotion effect

I. Introduction

The Yangtze River Delta (YRD) region has been one of the most dynamic regions in China in terms of economic development, openness, and innovation, as well as

the intersection of the "One Belt, One Road" and the Yangtze River Economic Belt, which is strategically vital for China's modernization and all-round opening up to the outside world. The General Secretary announced at the first China International Import Expo that the Yangtze River Delta region would be supported and elevated to a national strategy, marking a new stage of development for the Yangtze River Delta region. The Outline of the 14th Five-Year Plan and 2035 Vision for National Economic and Social Development of the People's Republic of China, released in 2021, once again reaffirmed that the Yangtze River Delta is the first place to accelerate the creation of a leading high-quality development echelon, aiming at international advanced science and innovation capabilities and industrial systems, accelerating the G60 science and innovation corridor of the Yangtze River Delta and the industrial innovation belt along Shanghai and Nanjing, improving the global resource allocation capability of the Yangtze River Delta and its radiation-driven ability to the whole country, and striving to enhance the level of integrated development of the Yangtze River Delta.

After years of grinding development, the comprehensive development level and synergy show that the Yangtze River Delta region has formed a better synergistic development situation (Guo Hubin and Qi Yuan, 2018). Since the Yangtze River Delta integration was upgraded to a national strategy in November 2018, the GDP of the Yangtze River Delta region has grown from 22.1 trillion yuan in 2018 to 27.6 trillion yuan in 2021. the per capita GDP of the Yangtze River Delta region reached 117,300 yuan in 2021, which is already nearly twice the level of GDP per capita of middle and high-income countries in 2020 (Shougui Luo, 2022). However, compared with Beijing-Tianjin-Hebei and the Pearl River Delta, the level of integrated development and market integration in the Yangtze River Delta is still low, the industrial layout is unreasonable, and the pressure for transformation and upgrading is high (Li Lei, 2016; Huang Zhengxue et al., 2018; Liu Zhiyan et al., 2022), and the level of integration of factor markets lags far behind that of commodity markets (Yang Fenghua et al., 2012). In addition to economic factors, various institutional barriers are essential factors

contributing to the distorted development of YRD integration (Liu, 2019). The uneven development within the Yangtze River Delta region, the severe phenomenon of "vassal economy," the stagnation of market integration and industrial division of labor, the difficulty of synergistic development between mega-cities and super-small cities, the inadequate coordination mechanism of regional interests and the current governmental assessment system, and the failure of Shanghai's core leading role to give full play to the Yangtze River Delta integration are all severe constraints to the development level (Tan Dingnan, 2016; Zhang Xueliang et al., 2018; Sun Bindong, 2022). Therefore, only by optimizing the industrial layout, strengthening the coordination of policies and interests within the region, deepening the division of labor and cooperation, and enhancing the capacity for collaborative scientific and technological innovation can we achieve a higher level of integrated development in the Yangtze River Delta (Wang Zhen, 2020; Chen Wen et al., 2021; Friday 7, 2022).

Given the economic development level, industrial layout, technology level, and market scale of the Yangtze River Delta, the Yangtze River Delta region can fully realize the division of labor and complementary advantages of industries by reducing inter-provincial trade costs and developing inter-provincial trade to enhance the level of integrated development. Then, as a critical factor affecting the inter-provincial division of labor and cooperation in the Yangtze River Delta, the actual situation and evolution trend of inter-provincial trade costs become an essential starting point for research. To this end, this paper intends to measure the inter-provincial trade costs of the Yangtze River Delta and examine the evolution of inter-provincial trade costs and their effects on the development of inter-provincial trade in the Yangtze River Delta to provide a new research perspective and policy ideas for the integrated development of the Yangtze River Delta. To this end, this paper will try to measure the inter-provincial trade costs of the Yangtze River Delta based on the perspective of value-added trade to portray the actual situation and evolution trend of inter-provincial trade costs of the Yangtze River Delta under the division of labor in

global value chains and analyze the promotion effect of inter-provincial trade costs of the Yangtze River Delta on inter-provincial trade development of the Yangtze River Delta accordingly, to provide a reference for the establishment of a unified market, industrial restructuring, transformation and upgrading of the Yangtze River Delta and the realization of high-quality integrated development goals. It also provides a reference for establishing a unified market, industrial restructuring, transformation and upgrading, and achieving high-quality integrated development.

Secondly, the inter-provincial trade costs calculated from bilateral decomposition are used to reveal the status of industrial integration and trade patterns within the Yangtze River Delta to explore the inherent obstacles to deepen the optimization of industrial layout and value chain division of labor cooperation within the Yangtze River Delta.

II. Literature Review

As an essential factor influencing trade development, trade costs are closely related to formulating and implementing many trade policy measures (Anderso et al., 2003). In the early stage of trade development, trade costs are mainly determined by transport costs, tariffs, and simple non-tariff barriers and are measured by using directly observable or quantifiable data, such as distance, transport costs, or tariff costs (Hummels, 2001; Limao et al., 2001). The advantages of the direct measure are that it is simple and intuitive, and it is easy to know the level of trade costs based on the relevant metrics. However, with the development of trade, the types of non-tariff barriers are not only increasing but also more hidden; secondly, the components of trade costs are also increasing, and trade costs should include insurance costs, search, and matching fees, international settlement costs, institutional and regulatory costs, and language communication costs, in addition to traditional trade costs such as transportation costs and tariff costs, to make the products reach the final consumers. Again, it is difficult to collect data to measure traditional trade

costs, and there are also statistical inconsistencies in the data of different countries and sectors. The existence of these problems makes the direct measurement method not well applied to the measurement of trade cost problems.

To effectively overcome the drawbacks of the direct measurement method, many scholars have started to adopt the indirect measurement method and have conducted multidimensional exploration for this purpose. Indirect measures are mainly divided into the price index method (Parsley and WEI, 2001), the regression method (McCallum, 1995; Hummels, 2001), and the inverse trade cost method using trade flows (Anderson et al., 2003; Novy, 2006; Chen et al., 2009). Initially, the gravity model in the regression method was more widely used (Limao et al., 2001; Francois et al., 2007). However, the gravity model in the regression method is premised on the strict assumption of partial equilibrium between the two countries, which tends to omit some critical variables. For this reason, Anderson et al. (2003) introduced multilateral resistance factors for correction. They constructed a static multi-country general equilibrium model, which equipped the traditional gravity model with a micro basis and simplified the difficulty and method of measuring trade costs. On this basis, Novy introduced the iceberg-type bilateral trade costs and the symmetry assumption of successively eliminating trade costs so that the model application has a solid theoretical foundation. Since then, the principle of trade cost measurement based on the ex post backward extrapolation of trade flows has gradually become the primary paradigm in this research field (Chen et al., 2011; Jacks et al., 2011).

Regarding trade cost measurement studies, domestic scholars have also made a lot of exploration and attempts (Xu Deyou et al. 2010; Xu Tongsheng et al. 2011; Hu Zongbiao et al. 2013; Zhou Dan et al. 2015). However, it is infrequent to apply the measure of foreign trade cost to the study of measuring inter-provincial trade cost in China. Whether Sheng, Bin, et al. (2011) and Liu, Jian (2103) use border effects or price indices to study China's interregional trade costs, or Xing, Weibo and Li, Shantong (2009) use VAT data to study China's interregional trade costs, they only analyze a part of the influencing factors

of interregional trade costs. Xu Tongsheng et al. (2013) are the first to apply the trade cost measure based on the ex-post backward projection of trade flows to the study of China's inter-provincial trade costs and measure China's inter-provincial trade costs accordingly and find that China's inter-provincial trade costs gradually decline. Significant industry differences exist, with the manufacturing industry having the lowest trade costs. Pan, Wenqing, et al. (2017) also measured China's inter-regional trade costs based on this principle and using China's regional input-output data from 1997-2007. Still, the results showed that China's inter-regional trade costs were generally rising, and the decline was only offered in the period after WTO accession. Since traditional trade statistics cannot exclude a large amount of double counting due to the trade of intermediate products under the value chain division of labor, the inter-provincial trade costs measured using the traditional statistical caliber trade data tend to distort the trade costs. They cannot effectively portray the actual situation of inter-provincial trade costs. Because of this, Yuan Kaihua et al. (2019) attempted to extend the Novy (2013) model to a value-added trade perspective. They tried to measure and compare interregional trade costs using Chinese regional input-output data.

As can be seen above, the existing results on trade cost research mainly use traditional statistical caliber trade data for analysis, which cannot solve the problem of double counting in standard statistical caliber trade data under the division of labor in global value chains, and thus can hardly reflect the actual situation of trade costs. Therefore, this paper will extend Novy's (2013) gravity model from the perspective of value-added trade to objectively measure the inter-provincial trade costs in the Yangtze River Delta to truly reflect the industrial division of labor and trade patterns in the Yangtze River Delta as a whole and between provinces, and to provide helpful policy insights for the establishment of a unified market in the Yangtze River Delta and the realization of high-quality integrated development goals. Compared with the existing literature, this paper is a pioneering study in the following aspects: first, by expanding

the gravity model, the inter-provincial trade costs of the Yangtze River Delta are measured from the perspective of value-added, to objectively and genuinely reflect the inter-provincial trade costs of the Yangtze River Delta; second, the industry characteristics and division of labor pattern of inter-provincial trade costs of the Yangtze River Delta are dynamically analyzed; third, the data obtained by differential decomposition are used to reveal the role of inter-provincial trade cost factors in the development of inter-provincial trade in the Yangtze River Delta. The third is to use the data from the differential decomposition to reveal the contribution of inter-provincial trade cost factors in the development of inter-provincial trade in the Yangtze River Delta.

III. Rationale and Data Sources

(i) Measurement of Trade Costs under Value Added Statistics

The main parameters and descriptions above are summarized in Table 1.

Table 1. Parameters and descriptions

Parameters	Descriptions	Notes
x^{ij}	Nominal exports from country i to country j	$x^{ij} \geq 0$
x^{ji}	Nominal exports from country j to country i	$x^{ji} \geq 0$
x^{ii}	Domestic trade of country i	$x^{ii} \geq 0$
x^{jj}	Domestic trade of country j	$x^{jj} \geq 0$
y^{i}	Nominal income in country i	$y^{i} \geq 0$
y^{j}	Nominal income in country j	$y^{j} \geq 0$
y^{W}	World revenues	$y^{W} \geq 0$; $y^{W} = \sum_j y^j$
t^{ij}	Bilateral trade costs	$t^{ij} \geq 0$
t^{ii}	Domestic costs for country i	$t^{ii} \geq 0$
t^{jj}	Domestic costs for country j	$t^{jj} \geq 0$
τ^{ij}	Bilateral trade costs	$\tau^{ij} \geq 0$
σ	Alternative resilience	$\sigma \geq 1$
$\prod^{i}$	Price indices for country i	$\prod^{i} \geq 0$
P^{j}	Price index for country j	$P^{j} \geq 0$

Anderson and Van (2003) propose an indirect gravity model with micro-foundations for measuring trade costs based on the symmetry assumption.

$$x^{ij} = \frac{y^i y^j}{y^W}\left(\frac{t^{ij}}{\prod^i P^j}\right)^{1-\sigma} \quad (1)$$

Novy (2013) relaxes the assumption of trade cost symmetry by introducing $t^{ij} \neq t^{ji}$, so that (1) becomes (2) after correction.

$$\tau^{ij} = \left(\frac{t^{ij}t^{ji}}{t^{ii}t^{jj}}\right)^{\frac{1}{2}} - 1 = \left(\frac{x^{ii}x^{jj}}{x^{ij}x^{ji}}\right)^{\frac{1}{2(\sigma-1)}} - 1 \qquad (2)$$

The measure does not assume frictionless domestic trade and reflects why bilateral trade costs are higher than domestic trade costs. It can be seen from Equation 2 that if bilateral trade flows x^{ij} and x^{ji} increase relative to domestic trade flows x^{ii} and x^{jj}, bilateral trade becomes more accessible compared to domestic trade, which means decreases in the cost of bilateral trade τ^{ij}, and vice versa. Thus, the method allows for an indirect inversion of the magnitude of bilateral trade costs from observable trade flows and has become the primary paradigm for indirect measures of trade costs.

In the traditional inter-industry own division of labor and trade model, the measure can provide an adequate standard of trade costs. However, with the in-depth development of the global value chain division of labor and trade, the rapid growth of business in intermediate products such as parts and components has become the main feature of international value chain trade, leading to the prevalence of third-country (sectoral) effects and extensive double counting in bilateral trade statistics. To this end, this paper will introduce value-added trade data to improve and extend the above gravity model for measuring bilateral trade costs. This paper will illustrate the value-added accounting approach using a multi-country, multi-sector example and WWZ (2105). Based on the input-output relationship, it is obtained that:

$$X = AX + Y = (I - A)^{-1}Y = BY \qquad (3)$$

In equation 3, X denotes the matrix of aggregate output of each economy, Y represents the matrix of final demand of each economy, A denotes the matrix of the global production system, I denotes the unit matrix, and B denotes the inverse Lyontief matrix. A country's output originates from the demand for final goods in each economy. Assuming further that V is the value added coefficient

(the share of sectoral value added in production), the value added (Va) induced by the final demand of each sector in country j in each industry in country i can be expressed as:

$$Va^{ij} = V^{i1}X^{ij} = V^{i}B^{ii}(\sum_{k\neq i} A^{ik}X^{kj} + Y^{ij}) \quad (4)$$

Equation 4 well portrays the trade in intermediate component products along the GVCs and thus can be used to trace the natural source of value-added along the GVCs. Given this, the gravity model for bilateral trade cost measurement in the value-added perspective can be modified as

$$\overset{\Lambda}{\tau}{}^{ij}_{\hbar} = \left(\frac{Va^{ii}_{\hbar}Va^{jj}_{\hbar}}{Va^{ij}_{\hbar}Va^{ji}_{\hbar}}\right)^{\frac{1}{2(\sigma-1)}} - 1 \quad (5)$$

Based on the above principles, this paper will mainly use the value-added trade flows induced by the Yangtze River Delta and outer regions, as well as the inter-provincial demand of the Yangtze River Delta to make objective estimates of inter-provincial trade costs, and then portray the proper level and evolution of inter-provincial trade costs in the Yangtze River Delta.

(ii) Differential decomposition of inter-provincial trade flow promotion effects

Drawing on Xu Tongsheng (2013), this paper extends a multi-country general equilibrium trade gravity model on trade costs developed by Anderson (2003) to study the promotional effect of inter-provincial trade costs on development.

$$x^{ij} = \frac{y^{i}y^{j}}{y^{c}}\left(\frac{t^{ij}}{\prod^{i}P^{j}}\right)^{1-\sigma} \quad (6)$$

In Equation 6, the superscripts i, j, and c denote province i, province j, and China, respectively, x^{ij} denotes exports from province i to province j, y^{i} and y^{j} denote the nominal income of provinces i and j, respectively, y^{c} denotes the total size of the Chinese economy ($y^{c} = \sum_{j} y^{j}$), t^{ij} denotes the cost of bilateral inter-provincial trade in the Yangtze River Delta, and σ means the elasticity of substitution ($\sigma \geq 1$). $\prod^{i}$ and P^{j} denote the price indices of provinces i and j, respectively. By swapping the positions of the superscripts i and j in equation (3) with each other, we can get the formula for measuring

the size of exports from province j to province i.

$$x^{ji} = \frac{y^j y^i}{y^c}\left(\frac{t^{ji}}{\prod^j P^i}\right)^{1-\sigma} \quad (7)$$

Multiplying the left and right sides of Equations 6 and 7 give:

$$x^{ij}x^{ji} = (\frac{y^i y^j}{y^c})^2\left(\frac{t^{ij}t^{ji}}{\prod^i P^j \Pi^j P^i}\right)^{1-\sigma} \quad (8)$$

To facilitate the analysis of the impact of trade costs, economic growth, and multilateral resistance factors on the development of inter-provincial bilateral trade as measured by price indices, the logarithm of both sides of Equation 8 was continued to be taken simultaneously and then decomposed differentially. The specific results are as follows:

$$\Delta\, ln(x^{ij}x^{ji}) = 2\Delta\, ln\left(\frac{y^i y^j}{y^c}\right) + (1-\sigma)\Delta\, ln(t^{ij}t^{ji}) - (1-\sigma)\Delta\, ln(\Pi^i P^i \Pi^j P^j) \quad (9)$$

Dividing both sides of Equation 9 simultaneously by the first-order difference in bilateral trade flows $\Delta\, ln(x^{ij}x^{ji})$, we can obtain the following:

$$1 = \frac{2\Delta\, ln\left(\frac{y^i y^j}{y^c}\right)}{\Delta\, ln(x^{ij}x^{ji})} + \frac{(1-\sigma)\Delta\, ln(t^{ij}t^{ji})}{\Delta\, ln(x^{ij}x^{ji})} - \frac{(1-\sigma)\Delta\, ln(\Pi^i P^i \Pi^j P^j)}{\Delta\, ln(x^{ij}x^{ji})} \quad (10)$$

From equation (10), the three items on the right-hand side indicate the contribution of economic growth, changes in inter-provincial bilateral trade costs, and multilateral resistance to the growth of inter-provincial bilateral trade flows, with economic growth, falling inter-provincial bilateral trade costs and rising multilateral opposition to foreign trade having a positive contribution to the development of inter-provincial bilateral trade, and the opposite contribution effect being negative.

Using the indirect measure of trade costs formula $\tau^{ij} = \left(\frac{t^{ij}t^{ji}}{t^{ii}t^{jj}}\right)^{\frac{1}{2}} - 1 = \left(\frac{x^{ii}x^{jj}}{x^{ij}x^{ji}}\right)^{\frac{1}{2(\sigma-1)}} - 1$, we can get the expression for the calculation of $t^{ij}t^{ji}$. We substitute it into equation (10), and we can get conclude that:

$$1=\frac{2\Delta\ln\left(\frac{y^i y^j}{y^c}\right)}{\Delta\ln(x^{ij}x^{ji})}+\frac{2(1-\sigma)\Delta\ln(1+\tau^{ij})}{\Delta\ln(x^{ij}x^{ji})}-\frac{2(1-\sigma)\Delta\ln(\varphi^i\varphi^j)}{\Delta\ln(x^{ij}x^{ji})} \tag{11}$$

Given a large amount of double counting of trade flow data estimated by traditional statistical portals in the context of value chain division of labor, equation 11 is further extended using the value-added trade flow estimation approach of Equation 10 to obtain a measure of the contribution effect of each factor to inter-provincial trade development as follows:

$$1=\underbrace{\frac{2\Delta\ln\left(\frac{y^i y^j}{y^c}\right)}{\Delta\ln(Va^{ij}Va^{ji})}}_{\text{Economic growth affects}}+\underbrace{\frac{2(1-\sigma)\Delta\ln(1+\tau^{ij})}{\Delta\ln(Va^{ij}Va^{ji})}}_{\text{Trade cost-effectiveness}}-\underbrace{\frac{2(1-\sigma)\Delta\ln(\varphi^i\varphi^j)}{\Delta\ln(Va^{ij}Va^{ji})}}_{\text{Multilateral resistance effect}} \tag{12}$$

In equation 12, the promotion effects of the first two items on the right side of the equation on interprovincial trade development can be directly calculated from the relevant data, and the third multilateral resistance factor of foreign trade can be obtained through the value of the residual term.

(iii) Data sources

For the measurement of inter-provincial trade costs and value-added trade flows at the overall and inter-provincial levels of the Yangtze River Delta, this paper uses the 2012, 2015, and 2017 China Inter-Provincial and Inter-Regional Input-Output Tables. This paper also selects the GDP data published in the China Statistical Yearbook for 2012, 2015, and 2017 as the nominal income or economic size of the three provinces and one city in the Yangtze River Delta. For the value of the elasticity of substitution (usually 5-10), this paper still follows Novy (2013) and continues to set its value to 8.

IV. Levels of inter-provincial trade costs in the Yangtze River Delta and their evolutionary trends

(i) Overall inter-provincial trade costs of the Yangtze River Delta provinces/municipalities

Table 2. Overall inter-provincial trade costs of provinces/municipalities in the Yangtze River Delta under the value-added statistics

Unit: %

Province/ municipality	2012	2015	Change rate	2017	Change rate
Jiangsu	20.45	19.13	-6.46	17.74	-7.24
Shanghai	19.19	21.54	12.25	24.40	13.26
Zhejiang	22.84	20.64	-9.60	17.88	-13.40
Anhui	22.44	16.83	-24.98	28.76	70.87

According to Table 2, among the overall inter-provincial trade costs of the provinces/cities in the Yangtze River Delta measured under the value-added statistics, the lowest overall inter-provincial trade costs of the provinces/cities in the Yangtze River Delta in 2012, 2015, and 2017 are Shanghai, Anhui, and Jiangsu, whose trade costs are 19.19%, 16.83%, and 17.74%, respectively; while the highest overall inter-provincial trade costs of the provinces/municipalities in the Yangtze River Delta in the same period In the same period, the highest inter-provincial trade costs in the Yangtze River Delta provinces/cities are Zhejiang, Shanghai, and Anhui, with the corresponding trade costs of 22.84%, 21.54%, and 28.76% respectively. According to the trend of trade cost changes shown in the table, the inter-provincial trade cost in Shanghai has been increasing during the examination period, with an increase of 12.25% compared to 2012 and 13.26% compared to 2015. Inter-provincial trade costs in Jiangsu and Zhejiang provinces have decreased during the examination period. The decrease in inter-provincial trade costs in Jiangsu province was 6.46% and 7.24% in 2015 and 2017, and the reduction of inter-provincial trade costs in Zhejiang province was 9.60% and 13.40% in the same period. During the examination period, Anhui's inter-provincial trade costs, on the other hand, showed a trend of first will then rise, with a decrease of 24.98% in 2015 and a rise of 70.87% in 2017.

Compared with Xu Tongsheng et al. (2103) findings, the overall inter-provincial trade cost levels of all provinces/municipalities in the Yangtze River Delta have declined substantially compared to the earlier period. This is mainly

due to the deep integration of the Yangtze River Delta into the global value chain division of labor and the increasing degree of opening up to the outside world, forcing the domestic market to open up gradually as well, and the development of introduction up to the outside world has caused the domestic market integration to improve (Huang, Jiu-Li, 2011; Fan, Hen-Shan, 2012). From within the Yangtze River Delta, Shanghai's overall inter-provincial trade costs are on the rise, mainly because Shanghai has the fastest economic structural transformation and the highest proportion of service industries among the three provinces and one city in the Yangtze River Delta. Given the unique nature of service industries, its inter-provincial division of labor and cooperation is bound to lag behind manufacturing. All these negatively impact the value chain division of labor and cooperation between Shanghai and other provinces in the Yangtze River Delta, leading to a magnitude of sustained increase in trade costs. Shanghai, as the center of the economic development of the Yangtze River Delta, should play a more excellent leading and radiating role, significantly shifting the focus of cooperation from primary and secondary industries to tertiary industries to achieve the overall rapid development of the Yangtze River Delta regional economy.

(ii) Inter-provincial trade costs at the sectoral level of provinces/municipalities in the Yangtze River Delta

According to the results in Table 3, there are significant differences in inter-provincial trade costs at the sectoral level among provinces/municipalities in the Yangtze River Delta. In 2012, the sectors with the lowest inter-provincial trade costs at the sectoral level in Jiangsu, Shanghai, Zhejiang, and Anhui were S12, S12, S05, and S11, with trade costs of 3.51%, 1.68%, 2.03%, and 0.57%, respectively; the sectors with the highest inter-provincial trade costs are S28, with trade costs of 85.10%, 93.57%, 96.87%, and 87.59%, respectively. sectors were all S28, with trade costs of 85.10%, 93.57%, 96.87% and 87.59%, respectively. in 2017, the sectors with the lowest interprovincial trade costs in Jiangsu, Shanghai, Zhejiang, and Anhui became S11, S11, S07, and S02, with trade costs of

0.03%, 0.87%, 0.33%, and 1.73%, respectively; the highest trade sectors are all S35, with interprovincial trade costs of 131.75%, 116.55%, 102.27% and 119.15%, respectively. It can be seen that, on the one hand, the sectors with low trade costs are mostly manufacturing sectors, while the sectors with high trade costs are service sectors; on the other hand, there are large differences in inter-provincial trade costs at the sectoral levels of Jiangsu, Shanghai, Zhejiang, and Anhui, indicating that there is a significant complementary relationship of advantages at the sectoral levels of provinces/municipalities within the Yangtze River Delta. Provinces can vigorously develop their profitable industries, strengthen the division of labor and integration among sectoral levels in the region, improve the overall level of division of labor and cooperation, and strive to reduce inter-provincial trade costs to achieve high-level integrated development among sectors within the Yangtze River Delta region.

Although there is significant heterogeneity in inter-provincial trade costs at the sectoral level among provinces/municipalities in the Yangtze River Delta region, the inter-provincial trade costs of both sectors S11 and S12 in each province/municipality in the area are relatively low and belong to the dominant industrial industries in the Yangtze River Delta region. In contrast, the trade costs of both sectors, S28 and S35, are relatively high. This indicates that provinces/municipalities in the Yangtze River Delta have simultaneous competitive advantages or disadvantages in individual sectors. The provinces/municipalities in the Yangtze River Delta should focus on the sectors with higher trade costs, increase technological R&D and industrial upgrading, and focus on deepening the division of labor and integration with foreign or other domestic economic regions to realize the improvement of competitiveness and reduction of trade costs.

Table 3. Overall inter-provincial trade costs at the sectoral level in the YRD provinces under the value-added statistics

Unit: %

Department	2012				2015				2017			
	Jiangsu	Shanghai	Zhejiang	Anhui	Jiangsu	Shanghai	Zhejiang	Anhui	Jiangsu	Shanghai	Zhejiang	Anhui
S01	14.73	15.96	14.35	17.99	11.99	15.57	14.48	10.76	11.14	26.00	12.11	18.70
S02	-	-	-	-	-	-	-	-	-	-	-	1.73
S03	-	-	-	-	-	-	-	-	-	-	-	-

S04	–	–	–	–	–	–	–	–	–	–	–	–
S05	–	–	2.03	–	–	–	5.39	–	–	–	1.85	10.84
S06	12.85	20.05	17.11	23.16	10.77	20.13	13.87	16.98	16.91	33.03	15.45	26.08
S07	13.79	11.52	12.10	15.56	11.03	11.94	7.57	8.80	16.90	13.38	0.33	14.57
S08	32.85	30.14	31.31	33.07	27.54	26.75	23.06	24.58	38.24	38.98	27.68	47.73
S09	12.85	12.30	13.78	13.75	10.68	14.50	17.91	7.41	14.62	26.34	10.99	29.59
S10	9.27	5.37	7.29	7.46	8.32	8.55	3.71	1.45	4.32	7.56	1.21	9.05
S11	5.32	–	–	0.57	3.02	–	–	–	0.03	0.87	–	7.43
S12	3.51	1.68	2.44	6.34	2.71	–	1.08	0.41	0.15	2.97	–	9.98
S13	9.22	12.35	12.15	11.52	7.77	13.26	17.50	5.87	1.46	10.50	–	18.69
S14	6.70	5.93	3.62	1.38	3.17	10.65	8.17	–	2.50	–	5.47	10.22
S15	19.92	14.51	15.61	16.54	14.20	15.63	16.68	10.75	14.11	12.08	10.46	17.02
S16	20.27	22.44	26.17	24.15	19.97	21.38	18.90	18.30	27.36	29.82	21.86	20.18
S17	31.57	32.19	31.17	33.89	25.81	36.97	19.99	25.52	41.13	54.52	32.83	36.75
S18	35.50	29.35	33.61	39.22	29.76	23.22	25.52	31.12	20.57	28.40	19.73	31.83
S19	22.43	16.93	22.45	27.33	16.21	16.16	17.02	16.90	23.69	22.39	17.74	22.76
S20	16.01	8.92	22.01	22.49	14.79	14.67	13.49	13.49	17.29	23.72	8.75	15.41
S21	23.46	19.35	20.17	26.02	16.37%	13.00%	15.69%	15.65	20.29	21.13	3.39	11.09
S22	9.38	3.28	7.52	16.73	10.46	2.60	3.65	9.96	–	–	–	3.30
S23	–	–	–	–	-7.18	1.02	–	–	–	–	–	–
S24	–	–	–	–	0.25	–	–	–	3.91	9.71	–	10.64
S25	7.68	6.30	6.58	4.28	5.40	5.22	1.17	1.49	3.36	8.36	6.33	9.24
S26	6.68	11.05	9.97	7.30	4.54	11.38	7.06	3.59	–	14.56	8.69	4.10
S27	9.26	8.00	8.94	7.61	5.66	8.92	12.48	–	43.20	101.16	91.50	108.13
S28	85.10	93.57	96.87	87.59	77.21	86.50	81.81	77.48	10.45	17.93	8.33	21.31
S29	11.78	10.54	10.38	5.91	6.57	12.71	8.38	2.01	2.35	5.10	2.34	19.15
S30	5.04	–	7.08	3.17	3.77	1.89	7.18	–	8.88	23.27	14.97	19.55
S31	15.65	11.70	11.54	15.28	10.87	13.95	7.00	7.47	35.49	26.40	28.67	32.73
S32	17.13	10.79	19.07	19.02	14.60	12.01	15.07	14.81	4.02	11.56	5.01	14.54
S33	10.17	4.99	12.57	7.25	6.63	7.22	5.86	0.12	14.14	17.56	12.40	30.15
S34	33.20	12.72	37.62	31.59	23.98	12.76	22.93	12.63	2.93	9.42	–	14.47
S35	8.98	5.08	11.44	2.62	6.36	5.91	4.73	–	131.75	116.55	102.27	119.15
S36	23.28	24.90	25.36	28.18	21.45	25.58	30.44	18.65	8.47	11.39	27.34	26.71
S37	34.82	38.31	32.31	38.76	32.55	41.86	32.03	34.27	44.33	56.78	40.05	41.14
S38	14.31	8.85	15.92	11.54	13.47	8.36	14.48	7.02	9.44	5.12	10.57	13.39

S39	38.04	28.46	40.52	33.61	34.43	27.78	34.80	27.50	42.75	45.65	33.02	42.46
S40	65.39	69.07	52.33	75.39	62.43	64.89	28.72	63.05	104.75	103.95	91.92	116.46
S41	22.46	19.94	20.62	22.37	19.25	32.20	20.42	17.35	33.99	37.49	30.99	37.99
S42	39.12	23.39	40.17	54.36	37.57	26.13	40.27	28.09	21.69	32.40	20.92	70.89

Note: When the trade flow is 0, the overall inter-provincial trade cost at the sectoral level of the Yangtze River Delta provinces under the value-added statistics is not calculable, or data is missing and is indicated by "-."

（三）长三角省际双边贸易成本

(iii) Inter-provincial bilateral trade costs in the Yangtze River Delta

Table 4. Inter-provincial bilateral trade costs in the Yangtze River Delta under the value-added statistics

Unit: %

Province/municipality	2012				2015				2017			
	Jiangsu	Shanghai	Zhejiang	Anhui	Jiangsu	Shanghai	Zhejiang	Anhui	Jiangsu	Shanghai	Zhejiang	Anhui
Jiangsu	-	38.15	44.74	41.15	-	40.05	39.18	32.08	-	41.12	30.48	33.97
Shanghai	38.15	-	40.51	34.57	40.05	-	37.45	31.13	41.12	-	30.38	48.24
Zhejiang	44.74	40.51	-	41.78	39.18	37.45	-	31.79	30.48	30.38	-	42.68
Anhui	41.15	34.57	41.78	-	32.08	31.13	31.79	-	33.97	48.24	42.68	-

From the results shown in Table 4, it is clear that the inter-provincial bilateral trade costs in the Yangtze River Delta fluctuated significantly during the examination period, and there are significant differences in the absolute level and fluctuation trend of inter-provincial bilateral trade costs. From the fluctuation trend, Shanghai-Suzhou bilateral trade costs have been slightly increasing, and bilateral trade costs rose from 38.15% in 2012 to 40.05% in 2015 and further rose to 41.12% in 2017; Zhejiang and Anhui, Suzhou and Shanghai and Anhui bilateral trade costs all showed a fluctuation trend of first decreasing and then increasing, and bilateral trade costs respectively fell from 41.78% in 2012, 41.15% and 34.57% in 2012 to 31.79%, 32.08% and 31.13% in 2015, and then

rose to 42.68%, 33.97% and 48.24% in 2017; bilateral trade costs of Jiangsu, Zhejiang, and Shanghai-Zhejiang have been showing a decreasing trend, with bilateral trade costs falling from 44.74% and 40.51% in 2012 to 39.18% and 37.45% in 2015, and further decreased to 30.48% and 30.38% respectively in 2017.

In terms of absolute level, in 2012, the bilateral trade cost of Shanghai and Anhui was the lowest, with a trade cost of 34.57%; followed by the bilateral trade cost of Shanghai and Suzhou, with a trade cost of 38.15%; the highest trade cost was Zhejiang and Suzhou, with a high of 44.74%. Compared with 2012, the bilateral trade cost of Shanghai and Anhui is still the lowest in 2015, at 31.13%; the provinces with the highest bilateral trade cost have changed, with the most increased bilateral trade cost of Shanghai and Suzhou, at 40.05%. But in 2017, the bilateral trade cost of Shanghai and Zhejiang became the lowest, only 30.38%, while the bilateral trade cost level of Shanghai and Anhui was the highest, reaching 48.24%. It can be seen that not only the level of economic development, industrial structure, and regional complementarity are essential factors influencing the change of trade costs and the formation of the division of labor pattern, but also the economic distance caused by geographical location also has an essential impact on trade costs. Compared with other provinces, the distance between Shanghai and Anhui is the farthest, which becomes a necessary obstacle to decreasing bilateral trade costs between Shanghai and Anhui.

V. Promotional effects of inter-provincial trade costs on inter-provincial trade development in the Yangtze River Delta

(I) Promotional effect of inter-provincial trade cost on the overall inter-provincial trade development of the provinces/municipalities in the Yangtze River Delta

It is easy to know from equation 11 that trade flow is negatively related to the trade cost factor and positively associated with economic growth and multilateral resistance factors. This implies that financial increase, multilateral resistance rise, and trade cost decrease can effectively promote

the development of inter-provincial trade in the Yangtze River Delta.

According to Table 5, the growth rates of value-added trade export flows of each of Jiangsu, Shanghai, Zhejiang, and Anhui to other provinces/municipalities within the Yangtze River Delta during 2012-2017 are -13.25%, 27.15%, -21.72% and 28.16% in that order. In terms of the factors influencing the change in value-added trade flows, Jiangsu and Zhejiang have negative growth in value-added export trade flows, and the main controlling elements for the change are economic growth and declining trade costs. For Jiangsu, the contribution of economic growth is the largest, reaching 80.22%; the assistance of the decline in trade costs is the second largest, at 27.95%; the contribution of multilateral resistance to corrosion is the smallest, at 8.17%. For Zhejiang, where trade flows are also negative, the contribution of economic growth is 53.98%, the donation of falling trade costs is 35.99%, and the contribution of decreasing multilateral resistance is -10.03%.

For Shanghai and Anhui, the value-added export trade flows grew positively during the period under examination, with growth rates of 27.15% and 28.16%, respectively. Economic growth is also the main influencing factor for the development of inter-provincial trade in Shanghai and Anhui, with contribution rates of 80.91% and 112.56%, respectively; the contribution of the decline in multilateral resistance has the second highest impact on the trade flows in Shanghai (-74.32%), and the contribution of the reduction in trade costs has the second highest impact on the trade flows in Anhui (-83.25%); the influencing factors that contribute the least to the changes in trade flows in Shanghai and Anhui The factors contributing least to the changes in trade flows in Shanghai and Anhui are the decline in trade costs and the reduction in multilateral resistance, respectively, with their contributions of -55.32% and -70.69%, in that order.

Table 5. Decomposition of the effects of overall inter-provincial trade development in the Yangtze River Delta provinces/municipalities, 2012-2017

Unit: %

Region	The growth rate of	Contribution to income	Contribution to	Contribution of

	inter-provincial value-added trade flows	growth	declining trade costs	declining multilateral resistance
Jiangsu	-13.25	80.22	27.95	8.17
Shanghai	27.15	80.91	-55.23	-74.32
Zhejiang	-21.72	53.98	35.99	-10.03
Anhui	28.16	112.56	-83.25	-70.69

(II) Contribution effect of inter-provincial trade costs on inter-provincial bilateral trade development

To further understand the regional differences of inter-provincial trade development and its influencing factors among provinces/municipalities in the Yangtze River Delta, a differential decomposition of the growth of inter-provincial bilateral value-added trade flows in the Yangtze River Delta and the contribution rate of its related factors is made. The specific results are shown in Table 6. It is the largest, up to 335%. Suzhou-Shanghai and Anhui-Shanghai's bilateral value-added trade flows declined significantly with -43,51% and -45.65%, respectively. From the results of differential decomposition, there are significant differences in the factors that contribute most to the changes in bilateral value-added trade flows among provinces, among which economic growth factors contribute most to the changes of bilateral value-added trade flows of Shanghai-Suzhou, Shanghai-Anhui, Suzhou-Shanghai, Suzhou-Anhui, Zhejiang-Anhui, Anhui-Shanghai, Anhui-Suzhou, and Anhui-Zhejiang, with their corresponding contribution rates of 160.95%, 632.98%, 160.95%, 87.24%, 132.04%, 632.04%, and 632.65%, respectively. The corresponding contribution rates are 160.95%, 632.98%, 160.95%, 87.24%, 132.04%, 632.98%, 87.24%, and 132.04%, respectively; the factor of decreasing trade cost has the most significant contribution to the change of bilateral value-added trade flows of Shanghai, Zhejiang, Jiangsu, Zhejiang, Shanghai, and Zhejiang-Suzhou, with the corresponding contribution rates of 45.18%, 91.45%, 45.18%, and 91.45% respectively; the factor of decreasing

multilateral resistance has the most significant contribution to the change of bilateral value-added trade flows of Shanghai, Zhejiang, Shanghai, Anhui, Shanghai, and Zhejiang. Trade flows also have a specific contribution to the growth of trade flows, whose contribution rates are -17.73%, -399.60%, -17.73%, and -399.60%, respectively. In addition, as the bilateral trade costs of Shanghai-Suzhou, Shanghai-Anhui, Suzhou-Shanghai, Zhejiang-Anhui, Anhui-Shanghai, and Anhui-Zhejiang have increased instead of decreased during the period under examination, resulting in the inter-provincial trade cost factor becoming an obstacle to the development of bilateral trade.

The promotion effect of interprovincial trade development and its influencing factors in the Yangtze River Delta shows that the economic growth factor makes the most significant contribution to interprovincial trade development, and this result is consistent with the leading research findings at home and abroad (Novy, 2009; Xu, Tongsheng, et al., 2013). The contribution of inter-provincial trade cost factors to inter-provincial trade development in the Yangtze River Delta is much lower than that of economic growth factors, which is mainly due to the minimal decrease in inter-provincial trade costs in the Yangtze River Delta and the fact that some of them have increased instead of decreased, which limits the promotion effect of trade cost factors on trade development.

Table 6. Effect decomposition of inter-provincial bilateral trade development in the Yangtze River Delta, 2012-2017

Unit: %

Region	Inter-provincial trade growth rate	The contribution rate of income growth	The contribution rate of declining trade costs	The contribution rate of declining multilateral resistance
Shanghai-Jiangsu	207.68	160.95	-53.87	7.08
Shanghai-Zhejiang	335.00	37.09	45.18	-17.73
Shanghai-Anhui	112.76	632.98	-932.58	-399.60

Jiangsu-Shanghai	-43.51	160.95	-53.87	7.08
Jiangsu-Zhejiang	141.30	55.41	91.45	46.86
Jiangsu-Anhui	49.21	87.24	66.67	53.91
Zhejiang-Shanghai	133.55	37.09	45.18	-17.73
Zhejiang-Jiangsu	102.78	55.41	91.45	46.86
Zhejiang-Anhui	41.93	132.04	-12.72	19.32
Anhui-Shanghai	-45.65	632.98	-932.58	-399.60
Anhui-Jiangsu	100.57	87.24	66.67	53.91
Anhui-Zhejiang	41.42	132.04	-12.72	19.32

VI. Conclusion and Policy Suggestions

Given that the value-added statistical port data can solve the problem of duplicate statistics caused by a large amount of intermediate product trade in the global value chain division of labor to effectively reflect the actual situation of the worldwide value chain division of labor. To this end, this paper uses value-added statistics port trade data to objectively measure and analyze inter-provincial trade costs in the Yangtze River Delta. It uses the differential decomposition method to explore the contribution effect of inter-provincial trade costs to the growth of inter-provincial trade flows in the Yangtze River Delta. The study draws the following conclusions:

First, there are significant differences in the fluctuation trends of the overall inter-provincial trade costs of the provinces/municipalities in the Yangtze River Delta. During the examination period, the overall inter-provincial trade costs of Jiangsu and Zhejiang have been on a downward trend, with a decline of 2.71% and 4.96%; the overall inter-provincial trade costs of Anhui are on a trend of first going up and then rising, with an overall increase of 6.32%; the overall inter-provincial trade costs of Shanghai have been on an upward trend, with a rise of 5.21% in trade costs. Shanghai's overall inter-provincial trade costs are not only not the lowest in the Yangtze River Delta region, and trade costs are increasing rather than decreasing, a phenomenon that seems a bit bizarre. This is mainly due to the rapid transformation of Shanghai's industrial structure. The proportion of the service sector is significantly higher than the other three in the region. The regional inter-provincial division of labor and cooperation between the service sector lags behind the manufacturing sector.

Second, the difference in inter-provincial trade costs at the sectoral level of the Yangtze River Delta is mainly reflected in the relatively higher trade costs in the service sector. The difference in inter-provincial trade costs at the sectoral level among the provinces/cities in YRD is mainly reflected in the fact that the inter-provincial trade costs of the service sector as a whole are higher than those of the primary and secondary sectors, which is more in line with the pace of industrial structure transformation. The primary and secondary industries develop to a particular stage before gradually transforming into the tertiary sector, and the proportion of the service industry rises. This inevitably also leads to a lower degree of division of labor and cooperation in the service sector than in the primary and secondary industry sectors, and the inter-provincial trade costs in the service sector will naturally be relatively higher.

Third, the differences in inter-provincial trade costs in the Yangtze River Delta are also reflected in regional differences, i.e., significant regional differences in inter-provincial bilateral trade costs. At the beginning of the

examination period, Shanghai and Anhui bilateral trade costs are the lowest, and Zhejiang and Suzhou bilateral trade costs are the highest. However, at the end of the examination period, Shanghai and Anhui bilateral trade costs are the highest, and Shanghai and Zhejiang bilateral trade costs are the lowest. It can be seen that with the development and expansion of inter-provincial trade in the Yangtze River Delta, the degree of influence of the economic distance factor on trade costs has become more and more prominent. How to reduce the negative impact of trade cost reduction due to economic distance and thus promote the growth and integrated development of inter-provincial trade in the Yangtze River Delta is crucial.

Fourth, the decomposition of the promotion effect of inter-provincial trade cost factors on inter-provincial trade development in the Yangtze River Delta using the difference decomposition method finds that the contribution rate of inter-provincial trade cost factors to inter-provincial trade development needs to be further improved. The contribution rate of economic growth factors to inter-provincial trade development is much higher than that of trade cost factors. Therefore, it is necessary to vigorously promote the rapid development of inter-provincial trade in the Yangtze River Delta by reducing inter-provincial trade costs to improve the level of integrated development of the Yangtze River Delta.

Based on the effective measurement of inter-provincial trade costs in the Yangtze River Delta using trade data of value-added statistics, this paper further analyzes the promotion effect of inter-provincial trade costs on inter-provincial trade development in the Yangtze River Delta using the difference decomposition method. It provides a new policy design idea and path reference for deepening the division of labor and cooperation in the value chain within the Yangtze River Delta and achieving a higher level of integrated development. First, the three provinces and one city in the Yangtze River Delta should give full play to the advantages of their respective industrial sectors based on the principle of complementarity, deepen the inter-provincial sectoral division of labor and cooperation, cut inter-provincial trade costs, and promote the

development of inter-provincial trade. Second, strengthen the division of labor and cooperation between Shanghai and the other three provinces, reduce the overall inter-provincial trade costs of Shanghai, give full play to the central role of Shanghai in the economic development of the Yangtze River Delta, enhance the radiation and driving effect of Shanghai's development on other three provinces, and promote the integrated development of the Yangtze River Delta. Finally, to address the problems of high inter-provincial trade costs in the service sector and lagging inter-provincial division of labor and cooperation in the service sector, provinces/municipalities in the Yangtze River Delta, especially Shanghai, should use digital technology and other means to accelerate digital transformation and innovation in the service sector according to the unique attributes of the service sector, to facilitate the inter-provincial division of labor and cooperation in the service sector, change the situation of generally high inter-provincial trade costs in the service sector and enhance the role of declining inter-provincial trade costs in promoting inter-provincial trade growth in the Yangtze River Delta.

Reference:

[1] Anderson JE， Van Wincoop E. Trade Costs [J] .Journal of Economic Literature，2004，42(3):691-751.

[2] Butter F A G， Mosch R H J. Trade， trust and transaction costs[R].Tinbergen Institute Discussion Paper,2003.

[3] Chen N, Novy D. Gravity, Trade Integration, and Heterogeneity across Industries[J]. Journal of International Economics,2011,85(2): 206-221.

[4] Hummels D. Toward a geography of trade costs[R].Purdue University,2001.

[5] Jacks D S, Meissner C M, Novy D. Trade Booms, Trade Busts, and Trade Costs[J]. Journal of International Economics, 2011, 83(2): 185-201.

[6] Mc Callum, John. National Borders Matter: Canada—U.S.Regional Trade Patterns[J].American Economic Review,1995,85(3): pp. 615-623.

[7] Novy D. Is the Iceberg Melting Less Quickly? International Trade Costs after World War II[J]. Warwick Economics Research Paper, 2006.
[81] Novy D. Gravity redux: Measuring International Trade Costs with Panel Data[J]. Economic Inquiry, 2013, 51(1): 101-121.
[9] Poncet S. Measuring Chinese Domestic and International Integration[J].China Economic Review，2003，14(1):1-21.
[10] Hu Zongbiao and Wang Zuoli. Costs of international trade in services: international laws and Chinese experience[J]. Economic Research, 2013 Working Paper.
[11] Luo Shougui. Theory and Practice of Yangtze River Delta Integration from the Perspective of Collaborative Governance[J]. Journal of Shanghai Jiaotong University (Philosophy and Social Science Edition, 2022(4): 36-45.
[12] Pan Wenqing, Li Heqiang. Inter-regional trade costs in China: measurement and decomposition [J]. Quantitative Economic and Technical Economics Research, 2017(2):55-71.
[13] Sun Bindong. Theory and practice of high-quality integrated development of the Yangtze River Delta [J]. Academic Frontier, 2022(11):44-51.
[14] Xu Deyou, Liang Qi. Measurement and Analysis of China's Foreign Bilateral Trade Costs: 1981-2007[J]. Quantitative Economic and Technical Economics Research, 2010(1):119-139.
[15] Xu Tongsheng, Chen Jin, Xue Zhiyun. Measuring the trade costs of China's manufacturing industry[J]. China Industrial Economy,2011(7):15-25.
[16] Xu Tongsheng, Hong Yong, Tu Yuanfen, Huang Xianming. Measurement, effects, and determinants of inter-provincial trade costs in China after WTO accession[J]. Economic Review,2013(3):126-135.
[17]Yuan Kaihua, Peng Shuijun, Yu Yuan. Measurement and decomposition of inter-provincial trade costs in China from the perspective of value-added trade [J]. Statistical Research, 2019(2): 63-75.
[18] Wang Zhi, Wei Shangjin, Zhu Kunfu. Total trade accounting method: official trade statistics and the measurement of global value chain [J]. China Social

Science, 2015(9): 108-127.
[19] Friday Q. Dynamic evaluation of high-quality integrated development of Yangtze River Delta and its spatial characteristics analysis[J]. Economic System Reform, 2022(5): 67-74.
