# Supplementary material for: A study on the measurement of inter-provincial trade costs in Yangtze River Delta from the perspective of value-added trade and its promotion effect
Source: PLoS One. 2024 Mar 8;19(3):e0295654. doi: 10.1371/journal.pone.0295654 (PMC10923410; doi:10.1371/journal.pone.0295654)
Supplement: Attachment — Submitted filename: Response to Reviewers.docx [file pone.0295654.s002.docx]

**Dear Editors and Reviewers，**

Thank you for your letter and the reviewer's comments concerning our manuscript entitled " **A Study on the Measurement of Inter-provincial Trade Costs in Yangtze River Delta from the Perspective of Value Added Trade and its Promotion Effect" (ID: PONE-D-23-25349)**. Those comments are valuable and helpful for revising and improving our paper and the critical guiding significance of our research. We have studied the words carefully and have made a correction, which we hope meets with approval. The significant modifications in the paper and the response to the reviewer's comments are as follows:

Responds to the reviewer's comments:

**1. Response to comment:** The authors should further enhance the theoretical argument and contributions in the introduction section. This will help readers who are not familiar with the topic to better understand the research direction.

**Response:** We are very sorry for our negligence and have rewritten this part according to the reviewer's suggestion. The introduction section adds theoretical mechanism analysis, analyzing the specific mechanism of inter-provincial trade costs' impact on inter-provincial trade development from two aspects: transfer effect and creation effect.

**2. Response to comment:** The literature review should be enhanced by adding more literature studies and providing relevant citations. It can be further enriched with up-to-date studies to motivate the readers in the background and currency of the research subject.

**Response:** We are very sorry for our negligence and have rewritten this part according to the reviewer's suggestion. We have updated the literature and introduced preface theories and research from domestic and foreign scholars. The literature research section has added an introduction to the latest research results on thematic analysis.

**3. Response to comment:** The author should check and provide references/citations for all the sources used in the research.

**Response:** We are very sorry for our negligence and have rewritten this part according to the reviewer's suggestion. We have updated the references and formats according to the template requirements.

**4. Response to comment:** The methodology section needs improvements; The authors should further clarify and justify their choice of methods used. If possible, the author should provide additional pre/post estimation tests for the validations of the results.

**Response:** Considering the Reviewer's suggestion, We have refined and improved the push to process, and the derivation has become more precise and concise. The theoretical formula derivation section has been further refined, providing a step-by-step derivation process.

**5. Response to comment:** Presentation of the summary of descriptive statistics (Data) in a tabulated form is also recommended, which is missing in the article.

**Response:** This article did not conduct empirical regression analysis and did not conduct descriptive statistical analysis on the sample data.

**6. Response to comment:** The discussion on the results can be explained and enhanced with reference to previous literature studies and outcomes. It will provide further validity to the outcomes with regards to the literature outcomes.

**Response:** We are very sorry for our negligence and have rewritten this part according to the reviewer's suggestion. Further detailed explanations have been provided for some of the conclusions of the research results.

7. **Response to comment:** The language of the paper needs careful editing and proofreading to improve readability. The authors should recheck the format of the paper including the headings and subheadings. For instance, there is a Chinese Text under Section IV- (iii).

**Response:** Considering the Reviewer's suggestion, We have once again confirmed the grammar and format of the article to ensure its readability and researchability.

8. **Response to comment:** Please enhance the conclusion by further clarifying the limitations and future research directions.

**Response:** Considering the Reviewer's suggestion, a paragraph has been added at the end of the article to illustrate the shortcomings of this study and the direction for future improvement efforts.

Special thanks to you for your good comments. We appreciate the Editors/Reviewers' warm work earnestly and hope the correction will be approved.

Once again, thank you very much for your comments and suggestions.

Best wishes!

YangWang
