# Supplementary material for: A study on the measurement of inter-provincial trade costs in Yangtze River Delta from the perspective of value-added trade and its promotion effect
Source: PLoS One. 2024 Mar 8;19(3):e0295654. doi: 10.1371/journal.pone.0295654 (PMC10923410; doi:10.1371/journal.pone.0295654)
Supplement: Attachment — Submitted filename: Response to Reviewers.docx [file pone.0295654.s003.docx]

**Dear Editors and Reviewers，**

Thank you for your letter and the reviewer's comments concerning our manuscript entitled " **A Study on the Measurement of Inter-provincial Trade Costs in Yangtze River Delta from the Perspective of Value Added Trade and its Promotion Effect" (ID: PONE-D-23-25349)**. Those comments are valuable and helpful for revising and improving our paper and the critical guiding significance of our research. We have studied the words carefully and have made a correction, which we hope meets with approval. The significant modifications in the paper and the response to the reviewer's comments are as follows:

Responds to the reviewer's comments:

**1. Response to comment:** The authors have already added the limitations of the research in the conclusion section, but it needs to be brief and specific as some explanations can be part of the discussion section. I would suggest that the authors provide specific key points and recommendations briefly in the conclusion section.

**Response:** We are very sorry for our negligence and have rewritten this part according to the reviewer's suggestion.

**2. Response to comment:** Although the authors have addressed grammatical errors and enhanced the language of the article, it still needs proofreading and editing.

**Response:** We are very sorry for our negligence and have recheck all the paper according to the reviewer's suggestion.

Special thanks to you for your good comments. We appreciate the Editors/Reviewers' warm work earnestly and hope the correction will be approved. And also, we want to know the decision earlier if possible because we need this paper for our institutional promotion.

Once again, thank you very much for your comments and suggestions.

Best wishes!

Dr. Yang Wang

Shanghai Dianji University, China
